# Activation of pro-resolving pathways mediate the therapeutic effects of thymosin beta-4 during *Pseudomonas aeruginosa*-induced keratitis

**DOI:** 10.3389/fimmu.2024.1458684

**Published:** 2024-09-24

**Authors:** Yuxin Wang, Loveleen Banga, Abdul Shukkur Ebrahim, Thomas W. Carion, Gabriel Sosne, Elizabeth A. Berger

**Affiliations:** Department of Ophthalmology, Visual & Anatomical Sciences, Wayne State University School of Medicine, Detroit, MI, United States

**Keywords:** Tβ4, SPMs, keratitis, MΦ, phagocytosis, efferocytosis

## Abstract

**Introduction:**

Current treatments for bacterial keratitis fail to address the sight-threatening inflammatory host response. Our recent work elucidating the therapeutic mechanisms of adjunctive thymosin beta-4 (Tβ4) in resolving inflammation and infection in bacterial keratitis revealed modulation of effector cell function and enhanced bacterial killing. The current study builds upon the observed effects on effector cell function by investigating the impact of Tβ4 on specialized pro-resolving lipid mediator (SPM) pathways as they play a significant role in inflammation resolution.

**Methods:**

Using a well-established *in vivo* model of *Pseudomonas aeruginosa*-induced bacterial keratitis, we assessed key enzymes (5-LOX and 12/15-LOX) involved in SPM pathway activation, SPM end products (lipoxins, resolvins), and receptor levels for these mediators. *In vitro* validation using LPS-stimulated murine monocyte/MΦ-like RAW 264.7 cells and siRNA to inhibit Tβ4 and LOX enzymes was carried out to complement our *in vivo* findings.

**Results:**

Findings from our *in vivo* and *in vitro* investigations demonstrated that adjunctive Tβ4 treatment significantly influences enzymes and receptors involved in SPM pathways. Further, Tβ4 alone enhances the generation of SPM end products in the cornea. Our *in vitro* assessments confirmed that Tβ4-enhanced phagocytosis is directly mediated by SPM pathway activation. Whereas Tβ4-enhanced efferocytosis appeared to be indirect.

**Conclusion:**

Collectively, these findings suggest that the therapeutic effect of Tβ4 resolves inflammation through the activation of SPM pathways, thereby enhancing host defense and tissue repair. Our research contributes to understanding the potential mechanisms behind Tβ4 immunoregulatory function, pointing to its promising ability as a comprehensive adjunctive treatment for bacterial keratitis.

## Introduction

Keratitis is a debilitating condition characterized by inflammation of the cornea that can cause a wide range of symptoms, including eye pain, redness, blurred vision, sensitivity to light, corneal scarring, perforation, and endophthalmitis (1, 2). Keratitis-induced corneal opacification is among the leading causes of legal blindness worldwide, accounting for approximately 3.2% of all cases (3). *Pseudomonas aeruginosa* (PA) and *Staphylococcus aureus* (SA) are the two most prevalent bacteria associated with infectious keratitis (4, 5), with PA as the most common singular causative pathogen identified from major studies carried out in the US, UK, and Asia (6–11). As recommended by the American Academy of Ophthalmology, the preferred practice pattern is FDA-approved topical fluoroquinolone monotherapy (12). Although used off-label, fourth-generation ophthalmic fluoroquinolones (moxifloxacin, besifloxacin, gatifloxacin) are also effective against both Gram-positive and -negative bacteria, penetrate well into ocular tissue, and demonstrate better mutant prevention characteristics compared to older generations. While control of the bacteria is of utmost importance, antibiotics do not address the complete spectrum of pathological changes and healing processes involved. Treatment with corticosteroids does address the host response to an extent; however, their use remains judicious and unsubstantiated (13, 14). Hence, managing bacterial keratitis continues to be challenging, necessitating the exploration of alternatives or adjunct therapies to address aspects of extensive tissue damage and impaired healing.

Thymosin β4 (Tβ4) is a small, 43-amino acid polypeptide that has emerged as a promising adjunct therapy for bacterial keratitis due to its multifaceted influence on the host immune response and tissue repair processes. As a naturally occurring peptide, Tβ4 plays a crucial role in modulating cellular responses to injury and inflammation. The demonstrated ability of Tβ4 to promote wound healing, reduce inflammation, and regulate cell migration in various tissues, including the cornea (15–18), underscores its potential as an attractive candidate for enhancing inflammation resolution and promoting corneal tissue repair following infection. Our work establishing Tβ4 as a topical adjunct to ciprofloxacin for the treatment of bacterial keratitis has resulted in significantly improved disease outcomes. This improvement is characterized by reduced inflammatory mediators, enhanced bacterial killing, and activated wound healing in our experimental model of PA-induced keratitis (19, 20). Further, we have shown that Tβ4 regulates the infiltration of macrophages (MΦ) and polymorphonuclear neutrophils (PMN), two predominant immune cell types involved in the corneal response during keratitis (21, 22). Tβ4 has been shown to modulate their effector functions as well, including reactive oxygen and nitrogen production, NETosis, apoptosis, and efferocytosis. Despite establishing the therapeutic impact of Tβ4 on the inflammatory response, the mechanisms underlying its effects on inflammation resolution remain poorly understood.

Lipid mediators (LM) have critical roles in orchestrating the complex inflammatory response from its initiation to resolution. Proinflammatory leukotrienes (LTs), generated by 5-lipoxygenase (5-LOX) and 5-LOX-activating protein (FLAP), initiate and maintain inflammation (23). Specialized pro-resolving mediators (SPMs), including lipoxins, resolvins, protectins, and maresins, are endogenous LM that actively promote the resolution of inflammation, tissue repair, and the return to homeostasis (24–27). SPM biosynthesis predominantly depends on 12/15-LOX, a key marker of epithelial and mucosal pro-resolving pathway activity (28), with partial involvement of 5-LOX for lipoxin and resolvin biosynthesis, while maresin and protectin formation is independent of 5-LOX. SPMs exert their effects by binding and activating specific GPCRs, serving as “stop signals” that inhibit excessive inflammation and initiate the resolution phase (29). Previous work has highlighted the importance of a balanced axis of LOX pathways in inhibiting inflammation and promoting resolution and tissue restoration following corneal infection (28). Additionally, findings from a separate investigation suggest a potential, novel regulatory function of Tβ4 over the ‘resolution machinery’, including SPM enzymes, receptors, and end products (19).

During inflammation, MΦ upregulate the expression of receptors GPR18, FPR2, and ChemR23. When these receptors are activated by SPM binding, they enhance phagocytosis and efferocytosis (30). Enhanced efferocytosis of apoptotic PMN by MΦ further augments the production of SPMs (31). Given the pivotal role of SPMs in resolving inflammation and our previous findings regarding effector cell function, further exploration into how Tβ4 influences SPM pathways is warranted. Therefore, this study investigated the interplay between Tβ4, MΦ, and SPM pathways, revealing an immunoregulatory influence exerted by Tβ4 over SPM pathway activation. Furthermore, this activation augmented MΦ cellular function, leading to enhanced host response and tissue repair following corneal infection.

## Results

### Adjunctive Tβ4 treatment improves the corneal response through SPM pathway activation

#### SPM enzymes

We have previously shown that mice treated with Tβ4 exhibit a disease response comparable to those treated with PBS, emphasizing the importance of pathogen removal (19). Although ciprofloxacin-treated mice effectively cleared the bacteria, corneas still exhibited stromal edema, infiltrating cells within the anterior chamber, and a disrupted, detached epithelium. In contrast, mice treated with adjunctive Tβ4 showed minimal to no edema, significantly reduced inflammatory cell infiltrate, and a mostly intact epithelium, indicating activated wound healing and resolution of inflammation. To start investigating potential mechanisms underlying the pro-resolving effects of adjunctive Tβ4, the current study assesses the corneal response at 3 days p.i. This time point was chosen since significant improvements in clinical scores were observed, yet full restoration of corneal homeostasis, as seen at 5 days p.i., had not yet occurred, as previously shown (19).

**Figure 1:**
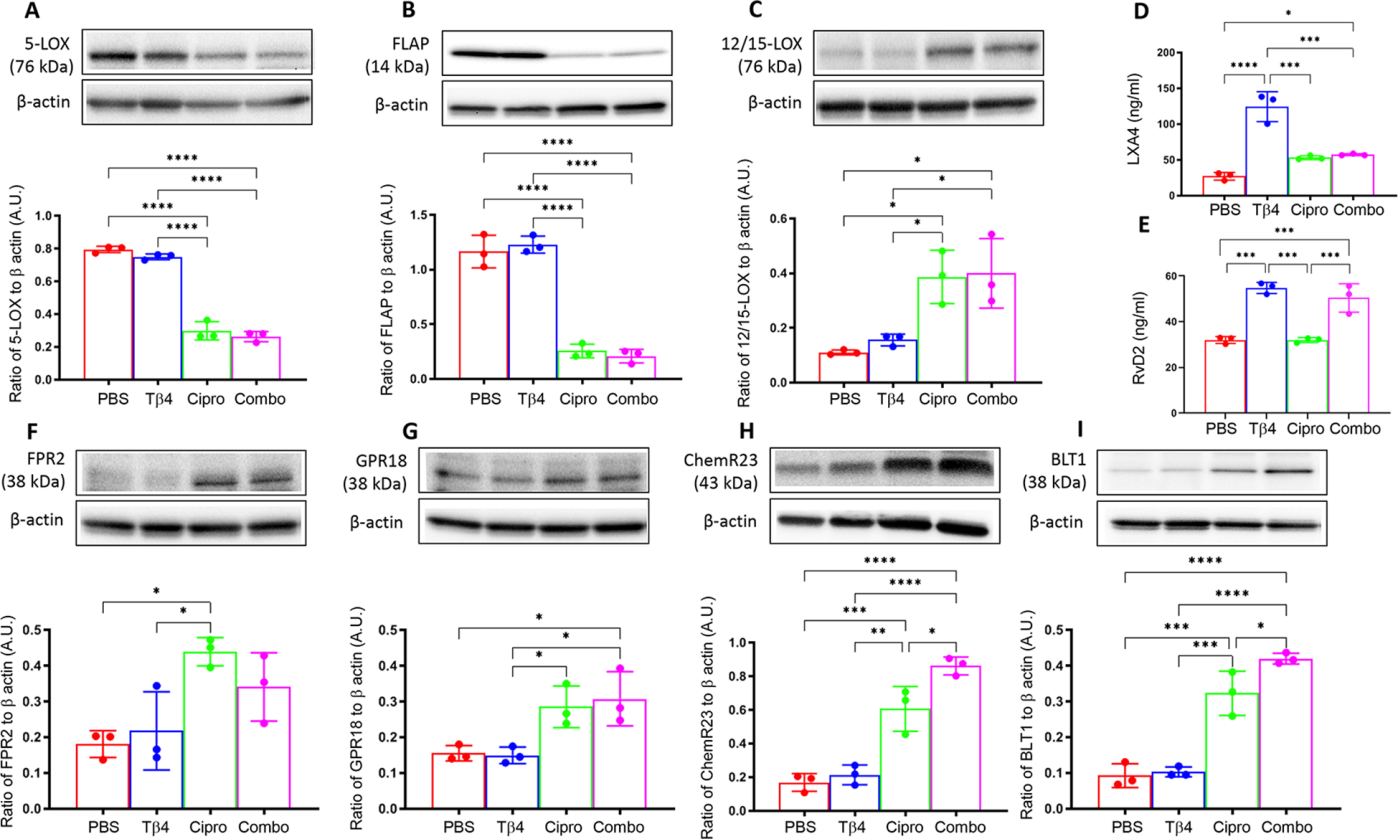
*In vivo* assessment of SPM enzymes, end products, and receptors following infection. Individual B6 mouse corneas were collected at 3 days p.i. SPM enzymes: 5-LOX **(A)**, FLAP **(B)**, and 12/15-LOX **(C)** were measured by Western blot. SPM end products: LXA4 **(D)** and RvD2 **(E)** were measured by ELISA. SPM receptors: FPR2 **(F)**, GPR18 **(G)**, ChemR23 **(H)**, and BLT1 **(I)** were measured by Western blot. Results of three independent experiments and have been normalized to β-actin with SD indicated. *p <0.05, **p < 0.01, ***p<0.001, ****p < 0.0001.

To begin, protein levels for 5-LOX (A), FLAP (B), and 12/15-LOX (C) were assessed by Western blot, as shown in **Figure 1**. These enzymes are involved in the metabolism of polyunsaturated fatty acids (PUFAs), particularly arachidonic acid, leading to the synthesis of lipid mediators. 5-LOX is mainly involved in the biosynthesis of pro-inflammatory leukotrienes. 5-LOX can also contribute to the generation of pro-resolving lipoxins and resolvins through alternative pathways. FLAP is an essential regulatory protein that associates with 5-LOX and facilitates its activation (32). Protein levels of both 5-LOX (A) and FLAP (B) were significantly upregulated in the corneas of PBS- and Tβ4-treated mice. However, both enzymes were significantly inhibited with ciprofloxacin and adjunctive Tβ4 treatments. 12/15-LOX is an important enzyme that catalyzes the oxidation of PUFAs, leading to the production of SPMs involved in inflammation resolution and tissue repair. While 12/15-LOX levels (C) were notably low in the corneas of PBS and Tβ4 treatment groups, enzyme levels were significantly elevated following ciprofloxacin and adjunctive Tβ4 treatments. These findings support a link between these treatments and the promotion of inflammation resolution and tissue repair through the activation of SPM pathways.

#### SPM end products

To continue exploring the influence of Tβ4 on SPM pathway activation, levels of two pro-resolving lipid mediator end products, LXA4 (D) and RvD2 (E), were assessed by ELISA in individual corneas at 3 days p.i. (**Figure 1**). Though LXA4 is derived from arachidonic acid metabolism and RvD2 is generated from the metabolism of omega-3 PUFAs, both are important signaling molecules that help to coordinate the resolution of inflammation. LXA4 is known to inhibit leukocyte recruitment, promote the resolution of inflammation, and enhance tissue repair processes. RvD2 plays a crucial role in resolving inflammation by inhibiting neutrophil infiltration, promoting efferocytosis, and stimulating tissue regeneration and repair. Corneal levels of LXA4 (D) were significantly elevated in mice treated with either Tβ4 alone or as an adjunct compared to the PBS control. In fact, LXA4 was significantly elevated following Tβ4 treatment compared to all other treatment groups (PBS, ciprofloxacin, and adjunctive Tβ4). Similarly, RvD2 levels (E) were significantly increased in both Tβ4- and adjunctive Tβ4-treated groups. These results highlight the potent effect of Tβ4, either alone or as an adjunct, in enhancing the biosynthesis of LXA4 and RvD2.

#### SPM receptors

FPR2, GPR18, ChemR23, and BLT1 receptors mediate the actions of lipoxins and resolvins. FPR2, or formyl peptide receptor 2, is expressed on immune cells and has been implicated in regulating inflammation, phagocytosis, and chemotaxis in response to LXA4 binding (33). GPR18, also known as N-arachidonyl glycine receptor, is expressed in immune cells, including Mɸ and PMN (34), and serves as a receptor for RvD2, contributing to its anti-inflammatory and pro-resolving effects (35, 36). ChemR23, also known as Chemokine-like receptor 1 (CMKLR1), is expressed on Mɸ, as well. RvE1 binding to ChemR23 promotes inflammation resolution and facilitates tissue repair (34). BLT1, or leukotriene B4 receptor 1, is a receptor for LTB4 and is expressed on various immune cells, including neutrophils and Mɸ, where it mediates inflammatory responses. However, BLT1 has been shown to bind RvD2 and contribute to inflammation resolution. In line with these observations, we further investigated all four receptors to gain insight into the extent of Tβ4’s influence over SPM pathway activation, as shown in **Figure 1**. Protein levels for FPR2 (F), GPR18 (G), ChemR23 (H), and BLT1 (I) were detected in individual corneas at 3 days p.i. by Western blot analysis. Although receptor levels were lowest in corneas of PBS and Tβ4 only groups, they were elevated after ciprofloxacin and adjunctive Tβ4 treatments. Notably, adjunctive Tβ4-treated mice showed significant differences in corneal levels of GPR18, ChemR23, and BLT1 receptors. These findings suggest that adjunctive Tβ4 not only modulates SPM biosynthesis but also influences SPM receptors, ultimately contributing to improved outcomes in bacterial keratitis.

### *In vitro* assessment of Tβ4 regulation over SPM pathway activation in Mɸ cell line

#### SPM enzymes

**Figure 2:**
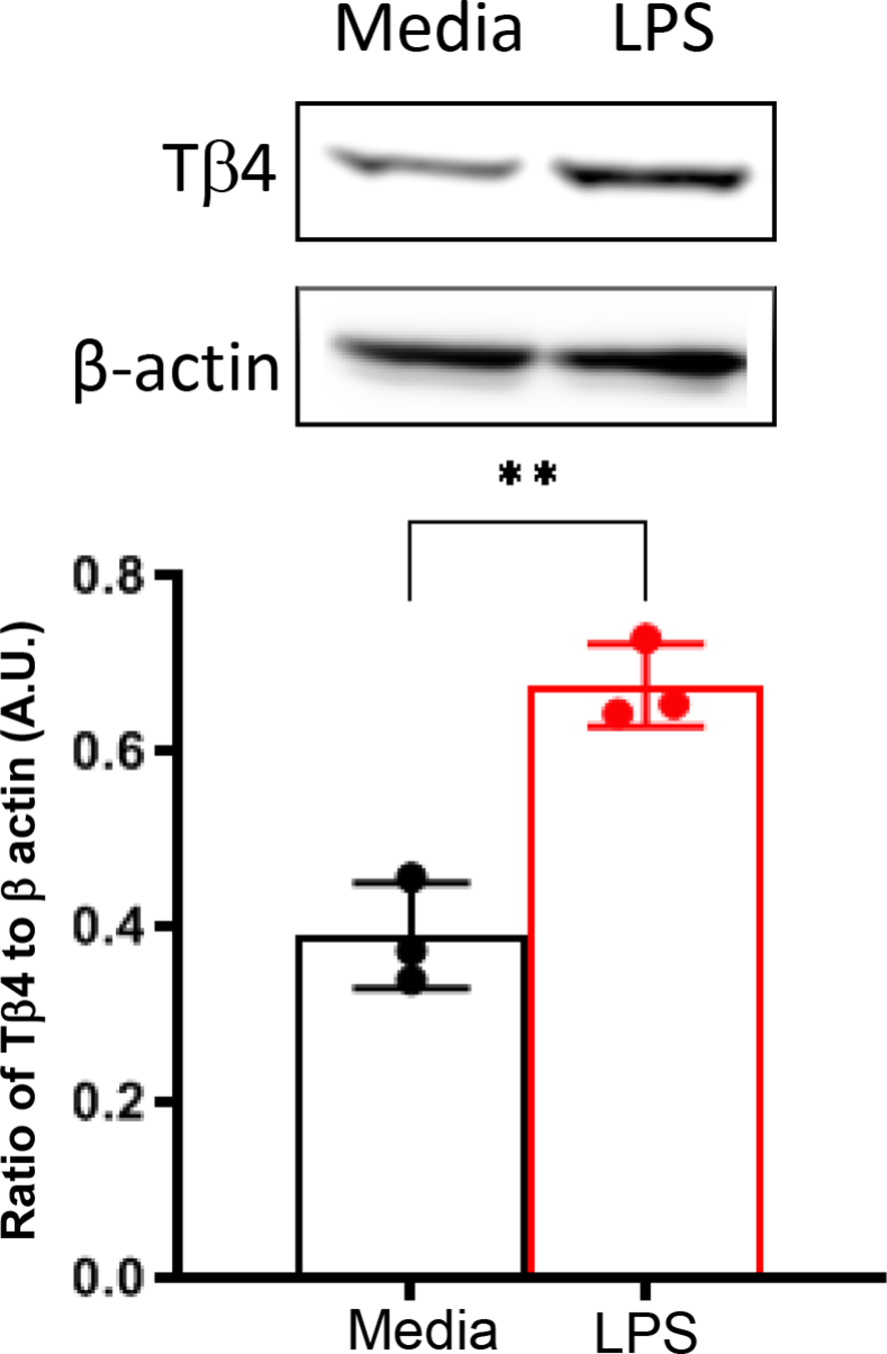
*In vitro* assessment of Tβ4 expression following LPS stimulation. Tβ4 was measured in media only and LPS-stimulated RAW 264.7 cells 24h after treatment using Western blot. Results are representative of three independent experiments and have been normalized to β-actin with SD indicated. ** p < 0.01.

*In vivo* assessments were complemented by *in vitro* investigation using a Mɸ-like cell line, RAW 264.7, to directly examine the impact of Tβ4 on SPM pathways at the cellular level. Given the enhanced responsiveness of Mɸ to Tβ4 in terms of their cellular function as observed *in vivo* (22), we initially measured Tβ4 levels in RAW 264.7 cells to confirm peptide expression. As shown in **Figure 2**, Mɸ not only express Tβ4 constitutively, but it is significantly up-regulated following LPS exposure.

**Figure 3:**
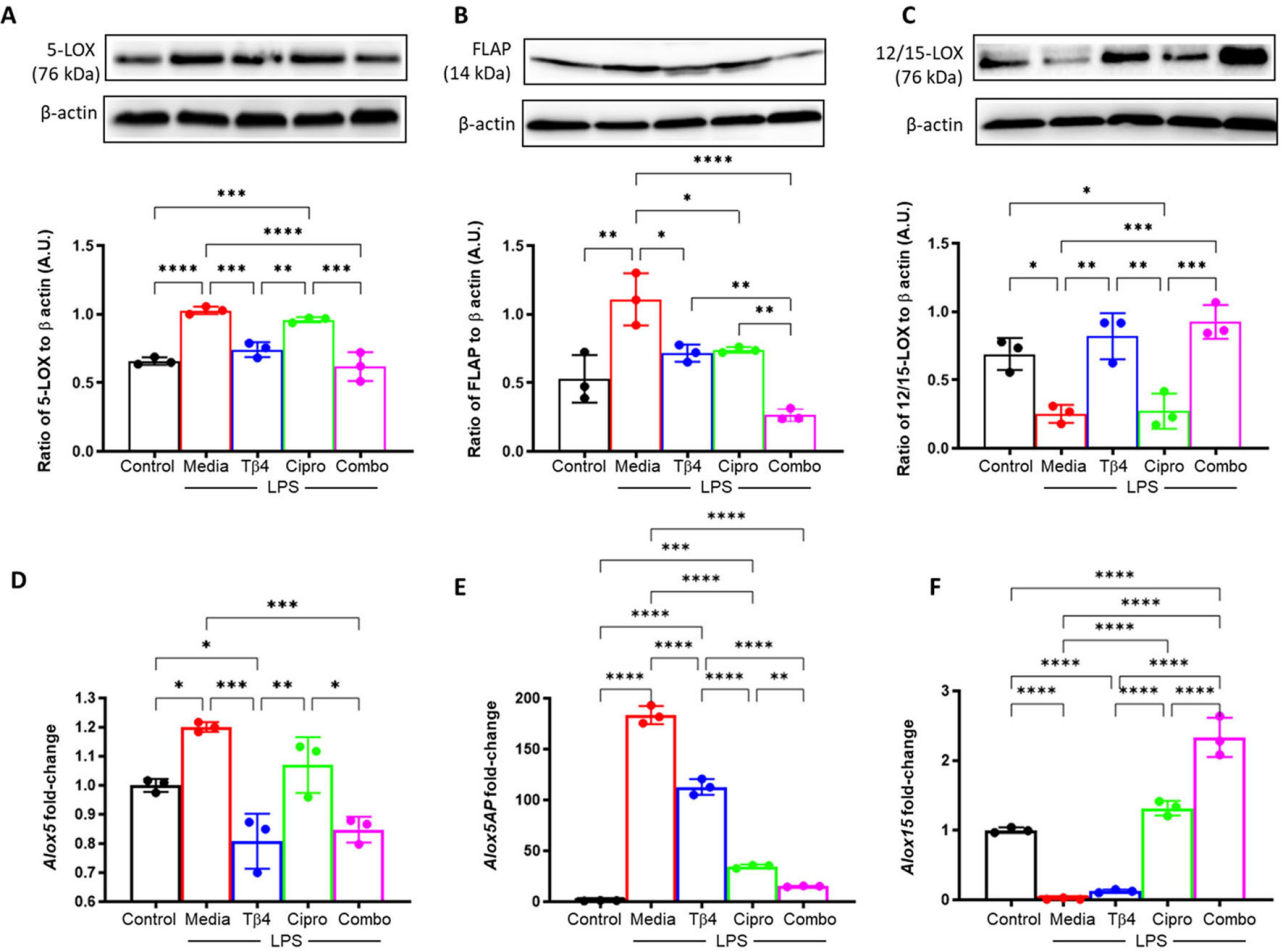
*In vitro* assessment of SPM enzymes following LPS stimulation. 5-LOX **(A, D)**, FLAP **(B, E)**, and 12/15-LOX **(C, F)** were measured in the media-, LPS-, Tβ4-, ciprofloxacin and combo-treated RAW 264.7 cells at 24 hours after LPS stimulation. Media only served as the negative control. The displayed data are representative of three independent experiments. Western blot results are shown as normalized values to β-actin with SD. RT-PCR data are reported as a relative fold-change for the target gene, normalized to β-actin with SD. *p < 0.05, **p < 0.01, 0.001, ***p < 0.001, ****p < 0.0001.

Next, it was examined whether the response to Tβ4 was directly related to SPM pathway activation. SPM enzymes (A – F) were determined at both the protein and mRNA levels subsequent to an LPS-induced inflammatory response (24h exposure), as shown in **Figure 3**. Consistent with the *in vivo* model, protein levels of 5-LOX (A) and FLAP (B) were significantly upregulated upon LPS stimulation yet significantly reduced following both Tβ4 and adjunctive Tβ4 treatments when compared to LPS. Though no differences were observed in 5-LOX after ciprofloxacin treatment, FLAP was significantly decreased compared to LPS. Conversely, the pro-resolving enzyme, 12/15-LOX (**C**), was significantly decreased following LPS stimulation, while both Tβ4 treatments resulted in significant increases versus LPS. No change in 12/15-LOX levels was noted following ciprofloxacin treatment. mRNA analysis largely mirrored these changes at the transcriptional level, with 5-LOX (D) and FLAP (E) reflecting protein levels. 12/15-LOX expression (F) was markedly low in the LPS and Tβ4 only groups but significantly increased with adjunctive Tβ4 compared to all other treatment groups.

#### SPM receptors

**Figure 4:**
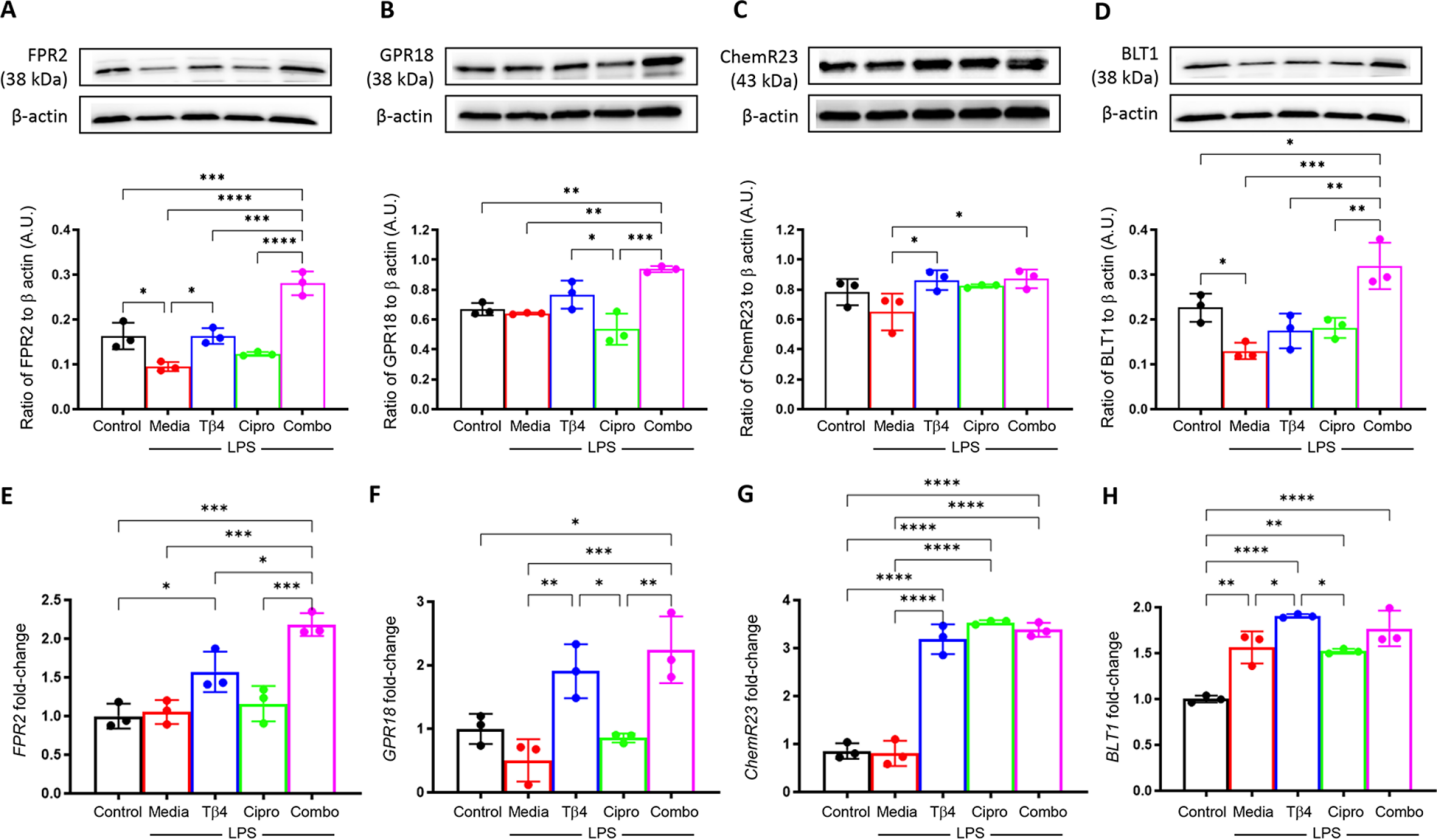
*In vitro* assessment of select SPM receptors following LPS stimulation. Levels of FPR2 **(A)**, GPR18 **(B)**, ChemR23 **(C)**, and BLT1 **(D)** in RAW 267.4 cells were determined by Western blot after 24 hours of stimulation and treatment. The results are shown as normalized values to β-actin with SD. mRNA levels of FRP2 **(E)**, GPR18 **(F)**, ChemR23 **(G)**, BLT1and **(H)** were quantified by real-time RT-PCR after 24h of stimulation and treatment. RT-PCR data are reported as a relative fold-change for the target gene, normalized to β-actin with SD. All experiments were performed in triplicate. *p < 0.05, **p < 0.01, ***p < 0.001, ****p < 0.0001.

Next, SPM receptors were similarly analyzed by Western blot (A – D) and real-time RT-PCR (E – H) to confirm the influence of Tβ4 treatment observed in the *in vivo* model of keratitis (**Figure 4**). Protein levels of FPR2 (A) and BLT1 (D) were significantly reduced, while no significant differences were observed with GPR18 (B) or ChemR23 (C) after LPS stimulation. Tβ4 treatment alone resulted in a significant up-regulation of FPR2 and ChemR23, while adjunctive Tβ4 treatment significantly increased all four receptors compared to LPS stimulation only. Ciprofloxacin treatment showed no influence on receptor levels following LPS stimulation.

Overall, trends were mixed between mRNA levels of SPM receptors compared to protein levels. No differences were observed after LPS stimulation when compared to media only controls for FPR2 (E), GPR18 (F), or ChemR23 (G), whereas BLT1 (H) was significantly increased. Both Tβ4 and adjunctive Tβ4 treatments resulted in significant up-regulation of transcript levels for FPR2, GPR18, ChemR23, and BLT1, except for adjunctive Tβ4 and BLT1 which showed no difference compared to LPS stimulation only. No differences were observed in receptor mRNA levels between ciprofloxacin treatment and LPS stimulation except for ChemR23, which was significantly increased. These findings suggest that Tβ4 stimulates SPM pathway activation via both enzymatic activity and receptors, which provides insight into the therapeutic mechanisms of Tβ4 regarding both inflammation and resolution.

### Tβ4-induced regulatory mechanisms of Mɸ effector cell function

#### Phagocytic activity

**Figure 5:**
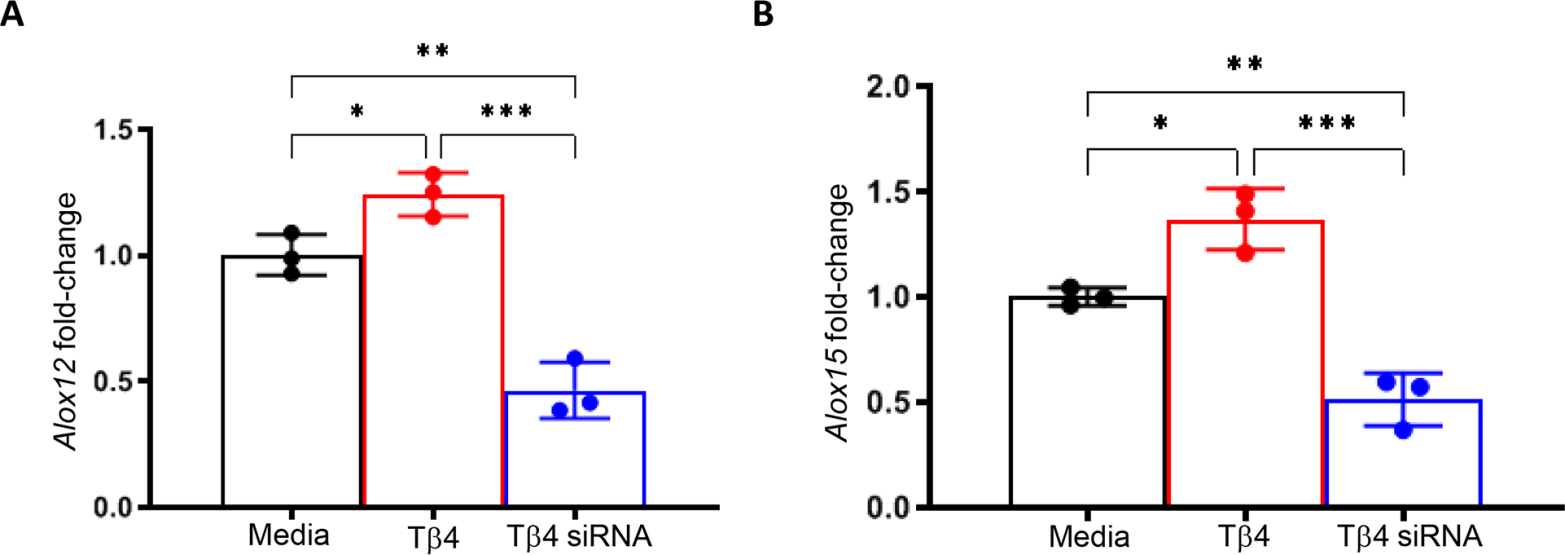
mRNA expression of LOX enzymes following Tβ4 stimulation and inhibition. The relative transcriptional level changes of 12-LOX **(A)** and 15-LOX **(B)** were measured in RAW 264.7 cells 72h after Tβ4 stimulation and inhibition by siRNA using RT-PCR. Results are representative of three independent experiments and have been normalized to β-actin with SD indicated. *p <0.05, **p < 0.01, ***p<0.001.

Building upon the finding that Tβ4 enhances MФ effector cell function in the cornea during infection, we hypothesized that this may be facilitated, in part, by SPM pathway activation. To begin, we confirmed the influence of Tβ4 on 12/15-LOX and 12-LOX mRNA expression in RAW 264.7 cells. It has been previously established that 12/15-LOX plays a regulatory role in the phagocytic function of mouse and human MФ (28, 37). However, it is known to have a primary 12-LOX activity as it generates 12-HETE and 15-HETE intermediate metabolites in a 3:1 ratio (28). Subsequently, 12-LOX was also examined to determine 15-LOX specificity. As shown in **Figure 5**, transcript levels for both enzymes were significantly increased when MФ were treated with Tβ4, which were then significantly decreased following inhibition of Tβ4 using siRNA. Dose responses for siRNA treatments are provided in **Supplementary Figure 1**. These data indicate that Tβ4 directly modulates the expression of SPM enzymes, supporting an SPM-dependent mechanism by which Tβ4 may enhance MФ function.

**Figure 6:**
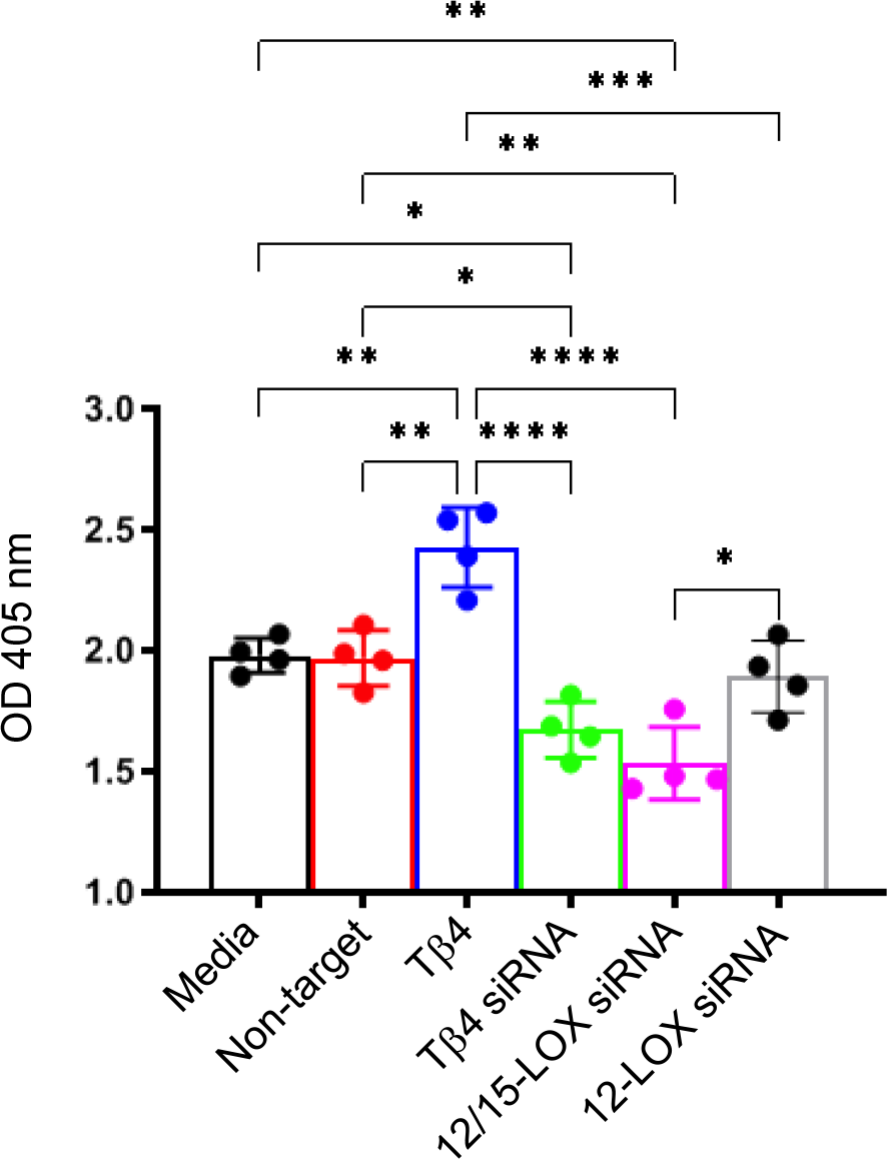
Phagocytosis activity was measured in Raw 264.7 cells. The influence of Tβ4 and LOX enzymes on the phagocytosis capacity of MΦ was evaluated using a zymosan-based phagocytosis assay. After a 48-hour incubation with Tβ4 and the corresponding siRNAs, the results are presented as mean absorbance values at 405 nm with SD. Each experiment was independently repeated four times. *p < 0.05, **p < 0.01, ***p < 0.001, ****p < 0.0001.

To further establish this regulatory influence, we next elucidated the effects of Tβ4, 12/15-LOX, and 12-LOX on phagocytotic activity in RAW 267.4 cells using a zymosan-based phagocytosis assay. As shown in **Figure 6**, Tβ4 treatment significantly increased phagocytosis compared to both the media-only and non-target negative control groups. Conversely, the phagocytic activity of MФ was significantly abrogated whether Tβ4 or 12/15-LOX was inhibited. However, 12-LOX inhibition was found to have no influence on phagocytosis, suggesting that 12-LOX does not play a direct role in the modulation of phagocytosis. The decreased phagocytic activity upon silencing Tβ4 and 12/15-LOX underscores their interlinked regulatory functions in this process. These results provide insight into how Tβ4 influences SPM pathways and how LOX pathways impact MΦ cellular functions during the resolution of inflammation.

#### MΦ efferocytosis

**Figure 7:**
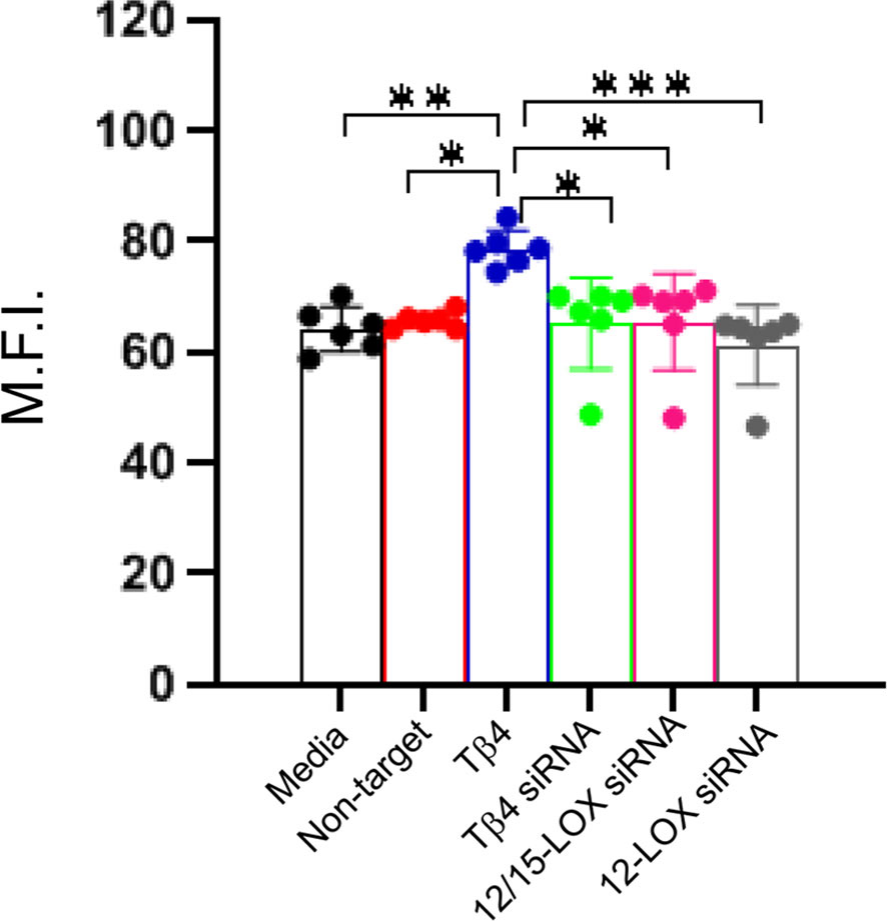
Efferocytotic activity measured in RAW 264.7 cells. The influence of Tβ4 and LOX enzymes on the efferocytotic capacity of MΦ was evaluated using the pHrodo Red Phagocytosis Assay. After RAW 264.7 cells were incubated with Tβ4 and the corresponding siRNAs for 48h, they were incubated with apoptotic HL-60 cells for 24h. Results are presented as mean fluorescence intensity (M.F.I.) with SD. Each experiment was independently repeated six times. *p < 0.05, **p < 0.01, ***p < 0.001.

Efferocytosis, the phagocytosis of apoptotic cells by MΦ, is critical to inflammation resolution. Previous work showed Tβ4 regulates MΦ efferocytotic activity (22), warranting an investigation of whether Tβ4 mediates its influence via SPM pathway activation. An efferocytosis assay was performed to examine the downstream effects of Tβ4 on MФ efferocytosis. **Figure 7** demonstrates that Tβ4 treatment significantly enhanced the efferocytotic activity of MФ following 24 h exposure to apoptotic leukocytes when compared to both the media-only and non-target negative control groups. Silencing Tβ4, 12/15-LOX, and 12-LOX markedly diminished the efferocytotic capabilities of MФ only when compared to the Tβ4 treatment group. In other words, there were no significant changes in the efferocytotic abilities of Tβ4, 12/15-LOX, and 12-LOX siRNA transfected MФs when compared to media-only and non-target negative control. Thus, though Tβ4 treatment increases the efferocytotic ability of MФ, this effect appears to be indirect.

## Discussion

PA-induced keratitis is a rapidly progressing corneal infection characterized by sight-threatening corneal opacification. For over 30 years, traditional ophthalmic fluoroquinolones have been commonly applied as an antibiotic monotherapy (38). However, the traditional treatment does not account for the uncontrollable host tissue inflammation that inflicts severe immunopathological damage to the cornea. Foundational research performed by Sosne et al. established that Tβ4 exhibits an anti-inflammatory role by preventing nuclear translocation of the NF-κB pathway (39). Studies from our lab further demonstrated that Tβ4 exerts an immunoregulatory effect by modulating the infiltration, activation, and function of MΦ and PMN in the context of PA-induced keratitis (21, 22). While understanding Tβ4’s influence on the proinflammatory limb of inflammation is important, it is equally important to study its impact on the resolution limb. Our previous work provided initial evidence supporting the hypothesis that Tβ4 enhances bacterial keratitis outcomes through functional SPM pathways (19). The current study reveals that adjunctive Tβ4 treatment enhances the host immune response by influencing key enzymes, end products, and receptors in SPM pathways. These findings highlight the dual nature of Tβ4 in regulating both the initiation and resolution phases of inflammation, leading to improved disease outcomes and restoration of tissue homeostasis in bacterial keratitis.

Previous work highlighted the crucial role of the well-balanced 5-/12-/15-LOX pathways in generating sufficient pro-resolving metabolites and maintaining immune homeostasis during bacterial keratitis (28). In addition, the ALX/FPR2 axis has been implicated in the anti-inflammatory and pro-resolving effects observed in PA-induced keratitis and *Aspergillus fumigatus* keratitis (40, 41). Furthermore, RvD1 has been shown to protect against ocular surface damage and reduce ocular allergic response in an experimental mouse model (42–44), while RvD2 has been detected in human tears and shown to exert physiologic functions in addition to resolution of inflammatory disease (45). RvD2 activates GPR18, leading to elevated cAMP levels, which increases intracellular calcium, resulting in stimulation of mucin secretion from conjunctival goblet cells (46). As such, RvD2 presents its potential as a novel treatment for dry eye disease. Similarly, RvE1 demonstrated anti-inflammatory and protective effects in various models of microbial keratitis and dry eye disease (47–49), acting through the activation of ChemR23 to block proinflammatory signals such as TNF-α-driven NF-κB activation (50). Another study by Arita et al. reported that RvE1 specifically binds the LTB4 receptor BLT1 and serves as a local damper of LTB4-BLT1 signals on PMN (51). Despite the growing interest in SPM pathways and their roles in specific inflammatory conditions, the precise mechanisms underlying the influence of Tβ4 on inflammation resolution and its correlation with SPMs in bacterial keratitis remain incompletely understood. In this study, we focused on investigating the impact of Tβ4 on the 5-/12-/15-LOX axis, as well as LXA4 and RvD2 and related receptors of SPM pathways in the cornea and MΦ, aiming to provide further insights into this interplay.

Understanding the influence of Tβ4 on SPMs and MΦ cellular function is crucial to understanding the corneal response to bacterial keratitis and the therapeutic mechanisms of Tβ4. However, an active infection greatly alters the impact of Tβ4, as observed in our *in vivo* infection model. This complexity makes it difficult to distinguish between the effects of Tβ4, cipro, and the cornea’s inherent healing ability once the pathogen is cleared. Therefore, *in vitro* studies are key to isolating Tβ4’s pro-resolving functions and demonstrate that the observed *in vivo* effects are, in fact, not cipro-driven.

SPMs enhance MΦ phagocytosis and efferocytosis, limit the production of pro-inflammatory cytokines, promote the production of anti-inflammatory cytokines, and induce an anti-inflammatory MΦ phenotype (24, 52). Our research uncovered that Tβ4 markedly upregulates the phagocytic activity of MΦ, which was notably reduced when Tβ4 expression was silenced. These findings align with previous work underscoring the role of Tβ4 in modulating the cellular functions of MΦ (22). Similarly, silencing 12/15-LOX, the most abundant LOX enzyme in the healthy cornea (53), significantly impaired the phagocytic capacity of MΦ. The role of 12/15-LOX in the resolution of inflammation is, in general, complex, as studies have shown conflicting results depending on the context of inflammation. One study revealed impaired inflammation resolution in 12/15-LOX-deficient mice, accompanied by impaired wound healing and increased post-inflammatory fibrosis (54). Another study demonstrated that the genetic removal of 12/15-LOX facilitated successful inflammation resolution following myocardial infarction (55). The influence of 12-LOX and 12/15-LOX is multifaceted in MΦ function, involving both direct and indirect mechanisms. In line with our observations, Miller et al. discovered that inhibition of 12/15-LOX activity significantly diminished actin polymerization during MΦ phagocytosis (56). Our findings further suggest that 15-LOX, not 12-LOX, is pivotal in modulating the phagocytic activity of MΦ.

During efferocytosis, phagocytes generate SPMs that act as autacoids to inhibit PMN activation, increase apoptotic cell expression of chemokine receptor CCR5 for chemokine clearance, and promote bacterial killing and efferocytosis by MΦs (27). We show that the addition of Tβ4 to MΦs significantly increased efferocytosis, but silencing Tβ4 produced no effect. This suggests that MΦ efferocytosis is enhanced by the addition of Tβ4, such that Tβ4 levels are above the homeostatic levels but not impacted by its absence. This could be because efferocytosis does not directly involve Tβ4 or there is another factor that can account for its lack. These findings are in contrast to our MΦ phagocytosis results, where phagocytosis was both enhanced by exogenous Tβ4 and impaired by a lack of Tβ4. Also, unlike our phagocytosis results, silencing 12/15-LOX or 12-LOX in MΦs did not affect efferocytotic activity. This finding suggests that Tβ4-enhanced efferocytosis is not directly mediated through LOX enzymes in the MΦ. Overall, there are differences between Tβ4’s effect on the SPM pathways as it relates to phagocytosis and efferocytosis, which might be attributed to the differing mechanisms of these processes.

However, the efferocytosis results should be interpreted with caution. Several *in vitro* studies have shown that MΦ treatment with SPMs can directly enhance efferocytosis (57–59). To explain our findings, we need to consider that SPM-mediated MΦ efferocytosis is not solely dependent on LOX enzyme activity. While they are critical in the production of SPMs, SPM effector function is dependent on the expression of the SPM receptors, such as ALX/FPR2 (lipoxins), ChemR23 (resolvins), and GPR32 (protectins), and enhanced by end products (LXA4 and RvD2). Moreover, there are various other components of the SPM machinery (i.e., receptors, end products, and/or efferocytosis-related genes) that may still be produced in the absence of LOX enzymes to drive efferocytosis. Of note, Dalli and Serhan showed that apoptotic PMNs elevate the production of SPMs (60). Moreover, it has been noted that SPM production involves transcellular biosynthesis and that apoptotic PMNs play a role in the transcellular production of SPMs during efferocytosis (60). Thus, the apoptotic neutrophil-like HL-60 cells used in our efferocytosis assay may have produced SPMs. This might explain why silencing the LOX enzymes did not reduce MΦ efferocytosis because SPMs released by HL-60 cells could bind to the SPM receptors on MΦs, activating SPM-driven efferocytosis.

While our study demonstrates the potential of adjunctive Tβ4 treatment in enhancing inflammation resolution in bacterial keratitis, it is necessary to acknowledge the limitations of this work. First, we used a murine model of PA-induced keratitis, which may not fully reflect the complexity of bacterial keratitis in humans. Second, our study focused on the effects of adjunctive Tβ4 treatment on the SPM pathway in MΦ but did not investigate other potential mechanisms, cell types, or cell-cell interactions that may contribute to the resolution of inflammation in bacterial keratitis. In conclusion, our study shows that adjunctive Tβ4 treatment modulates the expression of pro-inflammatory and pro-resolving enzymes involved in SPM pathways, promoting the production of pro-resolving lipid mediators, and modulating the expression of receptors for these mediators. In MΦ, Tβ4 enhances phagocytosis that directly involves SPM pathways, but the role of SPMs in Tβ4-enhanced efferocytosis appears to be indirect. Altogether, the current study provides an in-depth examination of the mechanisms behind inflammation resolution by Tβ4 adjunctive therapy through the SPM pathway. However, future studies are needed to further elucidate the influence of Tβ4 on SPM pathway activation, considering different effector cell types and other pathways involved in inflammation and infection to fully establish the clinical potential of Tβ4.

## Materials and methods

### Ethics statement

All experimental animals were housed and treated according to the guidelines set by the Wayne State University Institutional Animal Care and Use Committee. All experiments were performed according to the approved protocol (19–10–1312) and conformed to the Association for Research in Vision and Ophthalmology’s Statement on the Use of Animals in Ophthalmic and Vision Research.

### Experimental animal protocol

The *in vivo* infection model was carried out in female, 8-week-old C57BL/6 (B6) mice (The Jackson Laboratory; Bar Harbor, ME, USA). To initiate the infection, the left central cornea was wounded per established methods in the lab (19). Next, a 5-µL bacterial suspension containing 10^6^ CFU/µL of the cytotoxic *P. aeruginosa* strain ATCC 19660 (Manassas, VA, USA) was topically applied to the wounded cornea. The mice were then randomly divided into four treatment groups, including PBS as a positive control group, Tβ4 only (0.1%), ciprofloxacin or cipro only (0.3%), and the combination or combo Tβ4 (0.1%)/ciprofloxacin (0.3%). Treatments were administered topically (5 μL) 3× per day, beginning 24 hours after the initial (confirmed) infection and continued daily for the duration of the study. All animals were housed and treated in accordance with the guidelines set by the Wayne State University Institutional Animal Care and Use Committee (protocol 19-10-1312) and conformed to the Association for Research in Vision and Ophthalmology’s Statement on the Use of Animals in Ophthalmic and Vision Research.

### Cell culture and treatment

Murine-derived monocyte/MΦ RAW 264.7 cells (ATCC; TIB-71) were cultured in DMEM supplemented with 10% heat-inactivated FBS (Invitrogen Life Technologies, Carlsbad, CA, USA) and 100 U/mL penicillin/streptomycin at 37°C with 5% CO_2_. Prior to treatment, the cells were seeded in 6-well plates at a density of 0.2×10^6^ cells/well with a total volume of 2 mL. The cells were divided into four treatment groups: media only (positive control), Tβ4 only (0.1%), cipro only (0.03%), and combo Tβ4 (0.1%)/ciprofloxacin (0.03%). After reaching confluence, each group was stimulated with 25 µg/mL *P. aeruginosa*, serotype 10-derived LPS (Sigma-Aldrich; St. Louis, MO, USA). Media only without LPS stimulation served as the negative control. RAW 264.7 cells were used at passages 3-5 for this study.

### Protein analysis

Corneas from each treatment group were excised at 3 days p.i., then individually homogenized in RIPA buffer (Cell Signaling Technology, Danvers, MA, USA) with a protease and phosphatase inhibitor cocktail (Thermo Fisher Scientific, Waltham, MA, USA). RAW 264.7 cells were similarly collected after 24 hours of treatment and lysed in RIPA buffer and protease/phosphatase inhibitor. All samples were sonicated to ensure complete lysis, followed by centrifugation (12,000 RPM, 20 min). Supernatants were then collected and normalized to equal protein amounts using the BCA method.

For Western blot, the samples were separated onto 4-20% tris-glycine gels (Invitrogen; Carlsbad, CA, USA) and transferred to PVDF membranes. Following a 1h block with 5% non-fat milk in TBST (10 mmol/L Tris-HCl buffer, pH 8.0, 150 mmol/L NaCl, and 0.1% Tween 20) at room temperature, the membranes were incubated overnight at 4°C with primary antibodies targeting the specific proteins of interest. Subsequently, the membranes were incubated with goat anti-rabbit horseradish peroxidase-conjugated secondary antibodies (Thermo Fisher Scientific, Waltham, MA, USA) for 1h at room temperature. Chemiluminescence substrate kit (Thermo Fisher Scientific) was used to visualize the proteins. After image capture (Bio-Rad Molecular Imager, ChemiDoc XRS+), the expression levels of the target proteins were analyzed using Image Studio Lite software version 5.2 (LI-COR Biosciences, Lincoln, NE, USA), with normalizing to β-actin. Quantitative analysis was performed by measuring the band intensity of the target proteins relative to β-actin using densitometry. Each target protein was assessed at least three times with a representative blot shown. The antibodies used in the study were anti-5-LOX, anti-ChemR23 (1:500; Abcam, Cambridge, UK, Cat#ab169755, ab64881), anti-FLAP, anti-12/15-LOX, anti-GPR18 (1:1000; Abcam, Cambridge, UK, Cat#ab85227, ab23691, ab174835), anti-FPR2 (1:1000; Novus Biological, Centennial, CO, USA, Cat#NLS1878), anti-BLT1 (1:1000; Cayman Chemical, Ann Arbor, MI, USA, Cat#120114), anti-thymosin β-4 (1:1000; Millipore, Billerica, MA, USA, Cat#AB6019), and anti-β-actin (1:1000; Santa Cruz Biotechnology, Dallas, TX, USA, Cat#SC-47778).

For ELISA, samples were centrifuged for 5 min at 5000×g, and an aliquot of each supernatant was assayed in triplicate to measure the protein levels of LXA4 and RvD2 (Cayman Chemical, Ann Arbor, MI, USA) according to the manufacturer’s instructions. Absorbance readings were taken at 421 nm and 405 nm to calculate concentrations for each sample. The reported sensitivity of LXA4 and RvD2 assays are 52.4 pg/mL and 10 pg/mL, respectively. Data are presented as average pg/mL ± SD.

### Real-time RT-PCR

Total RNA was isolated from cultured cells for gene expression analysis using RNA-STAT 60 (Tel-Test, Friendswood, TX, USA) per the manufacturer’s instruction. RNA concentrations were then determined by spectrophotometry (260 nm). To construct the cDNA templates, 100 ng of total RNA was reverse transcribed, then amplified with the SYBR^®^ Green Master Mix (Thermo Fisher Scientific, Waltham, MA, USA) as per the manufacturer’s instructions, with the reaction mixture as previously described (28). Briefly, a 10-uL reaction mixture contained 5 µL of 2x SYBR Green PCR Master Mix, 1 µL diethylpyrocarbonate-water, 1 µL forward and reverse primers, and 2 µL cDNA (diluted 1:10). The primers were designed using Primer3 PCR v. 4.1.0 software and the sequences are listed in **Table 1**. The semi-quantitative real-time RT-PCR was carried out using the CFX Connect Real-Time RT-PCR Detection System (BioRad, Hercules, CA, USA). The changes at transcriptional level were measured using the relative standard curve method (61). The results are presented as the mean fold change ± SD after normalization with β-actin. The fold change was calculated by determining the ratio of the target gene expression in the experimental sample to that in the control sample (*in vivo*: uninfected, naïve cornea; *in vitro*: cells maintained in media only), normalized to β-actin.

**Table 1:** Nucleotide sequences of mouse primers used for PCR amplification.

Gene	Nucleotide Sequence	Primer
*Actb*	5’- ACT GGG AGA CAT GGA GAA G -3’ 5’- GTC TCC GGA GTC CAT CAC AA -3’	F R
*Alox5*	5’- ACT ACA TCT ACC TCA GCC TCA TT -3’ 5’- GGT GAC ATC GTA GGA GTC CAC -3’	F R
*Alox5ap*	5’-GCC GGA CTG ATG TAC CTG TT -3’ 5’-AGT TCT CAA AGT CGC TTC CG -3’	F R
*Alox15*	5’-TTT CTT AAT GGC GCC AAC CC -3’ 5’-CCC ATC AGG CTG CAA TTT CA -3’	F R
*Fpr2*	5’- CCT TGG ACC GCT GTA TTT GT -3’ 5’- CCC CAG GAT ACA AAG CTC AA -3’	F R
*Gpr18*	5’-CTT TGC CGT CCT GAT GCT AC -3’ 5’-GCG AAC ACT GCG AAG GTA AT -3’	F R
*Cmklr1*	5’-GGC TTT GGC TAC TTT GTG GA -3’ 5’-ATC TTG AAG GTG GCG ATG AC -3’	F R
*Ltb4r1*	5’- GCA TGT ATG CCA GTG TCC TG -3’ 5’- AAA AGA CAC CAC CCA GAT GC -3’	F R
*Gapdh*	5’-TAT GAC TCT ACC CAC GGC AAG T-3’ 5’-ATA CTC AGC ACC AGC ATC ACC-3’	F R

### Phagocytosis assay

The phagocytic capacity of RAW 264.7 cells was quantitatively assessed using the CytoSelect 96-well phagocytosis assay (Cell Biolabs, San Diego, CA, USA) in accordance with the manufacturer’s guidelines. To induce phagocytosis, enzyme-labeled zymosan particles, known for their potent stimulatory effect on this cellular process, were employed. Initially, RAW 264.7 cells were seeded at a density of 6 × 10^4^ cells/well. Subsequently, the cells were treated with siRNA for Tβ4, 12-LOX, or 15-LOX (On-TARGETplus Mouse Tmsb4x, Alox12 and Alox15; GE Healthcare Dharmacon, Lafayette, CO, USA) at a concentration of 50 nM for 48 h prior the addition of zymosan particles. The optimization of the siRNA concentrations was carried out prior to the experiments and is detailed in **Supplementary Figure 1**. Following the 48h of siRNA treatment, the cells were exposed to zymosan particles for one hour. The quantification of internalized particles was performed by measuring the absorbance at 405 nm using an automated plate reader.

### Efferocytosis assay

The efferocytotic ability of RAW 264.7 cells (effector cells) of apoptotic Human Leukemia HL-60 cells (target cells) was investigated by utilizing the Sartorius IncuCyte^®^ pHrodo Red Phagocytosis Assay (Essen BioScience, Ann Arbor, MI, USA) in accordance to the manufacturer’s guidelines.

Effector cell seeding density was optimized (10,000 cells/well) and resulted in 10-20% confluency at 24 hr in a 96 well-plate. The effector cells were incubated for 1h at 37°C to allow attachment before treatment with Tβ4 (0.1%) and transfection with Tβ4, 12-LOX, 15-LOX small interfering RNA (siRNA) (On-TARGETplus Mouse Tmsb4x, Alox12 and Alox15; GE Healthcare Dharmacon, Lafayette, CO, USA) at a concentration of 50 nM for 48h prior to adding the target cells. Optimization of the siRNA concentrations was performed per the manufacturer’s protocol and presented in **Supplementary Figure 1**. For comparison and to establish a baseline, siRNA (On-TARGETplus Non-Targeting Pool; GE Healthcare Dharmacon) was employed as a negative control.

Human Leukemia HL-60 cells (target cells) were routinely cultured in RPMI 1640 medium supplemented with 10% FBS. Cells from an exponentially growing culture at a density of 6 × 10^4^ cells/mL were induced to undergo apoptosis by treatment with 5 µM of camptothecin for a period of 18h. Apoptotic cells were then measured using RealTime-Glo™ Apoptosis assay (Promega Corporation, Madison, WI, USA). This assay detects caspase activity, a hallmark of apoptosis. Additionally, cells were stained with annexin V, which binds to phosphatidylserine residues translocated to the outer leaflet of the plasma membrane during early apoptosis. Camptothecin concentration optimization was conducted according to the manufacturer’s guidelines and is detailed in **Supplementary Figure 2**.

After 18 hr incubation with camptothecin, apoptotic HL-60 target cells were transferred to a 50 mL centrifuge tube and centrifuged for 7 min at 1,000 RPM. Media was aspirated off and the cell pellet was resuspended in 50 mL pHrodo^®^ Wash Buffer. Apoptotic target cells were centrifuged for 7 min at 1,000 RPM. Wash Buffer was aspirated off and the cell pellet was resuspended in pHrodo^®^ Labeling Buffer to a cell density of 1x10^6^ cells/mL. pHrodo^®^ Red Cell Labeling Dye was added to the target cells at a final concentration of 1 µg/mL. The cell suspension was incubated for 1h at 37°C. The target cell labeling dye suspension was centrifuged for 7 min at 1,000 RPM. The supernatant was aspirated off and the apoptotic target cells were resuspended in 50 mL RPMI. Target cells were harvested by centrifugation for 7 min at 1,000 RPM. The supernatant was aspirated off, and apoptotic target cells were resuspended in effector cell media (DMEM). 2.5 x10^5^ target cells were added to each well to give an efferocytotic ratio (target cells:effector cells) of 25:1 to allow efficient measurement of efferocytosis. Quantification of internalized target cells was detected by measuring the excitation/emission readings at 560/586 nm using an automated fluorescent plate reader at 24h after addition of target cells. Results are shown as mean fluorescent intensity (M.F.I.).

### Statistical analysis

Sample sizes were determined statistically prior to experimentation, taking into account a mortality rate of less than 5% based on previous work. Power analysis was conducted using parameters including a mean difference = 2, standard deviation = 1, significance level (α) = 0.05, power = 0.8, and a sample size ratio = 1. For the *in vivo* model, a minimum of n = 5 mice/group for each time point was used unless otherwise noted. All experiments were performed a minimum of three times to ensure reproducibility and representative data from a typical experiment are shown. Data are displayed as the mean ± SD unless otherwise stated. Statistical analysis was performed using one-way ANOVA and Bonferroni’s multiple comparison test (GraphPad Prism, San Diego, CA, USA). Results were considered significant at *p* < 0.05.

## Data Availability

The original contributions presented in the study are included in the article/**Supplementary Material**. Further inquiries can be directed to the corresponding author.
