# Supplementary material for: Activation of pro-resolving pathways mediate the therapeutic effects of thymosin beta-4 during Pseudomonas aeruginosa-induced keratitis
Source: Front Immunol. 2024 Sep 24;15:1458684. doi: 10.3389/fimmu.2024.1458684 (PMC11458456; doi:10.3389/fimmu.2024.1458684)
Supplement: Supplementary file 1 [file DataSheet1.docx]

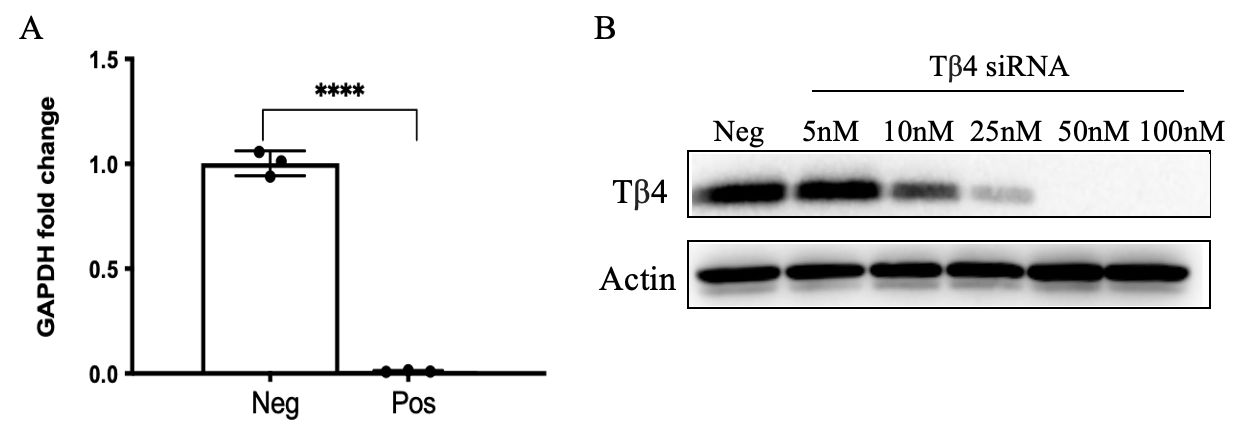


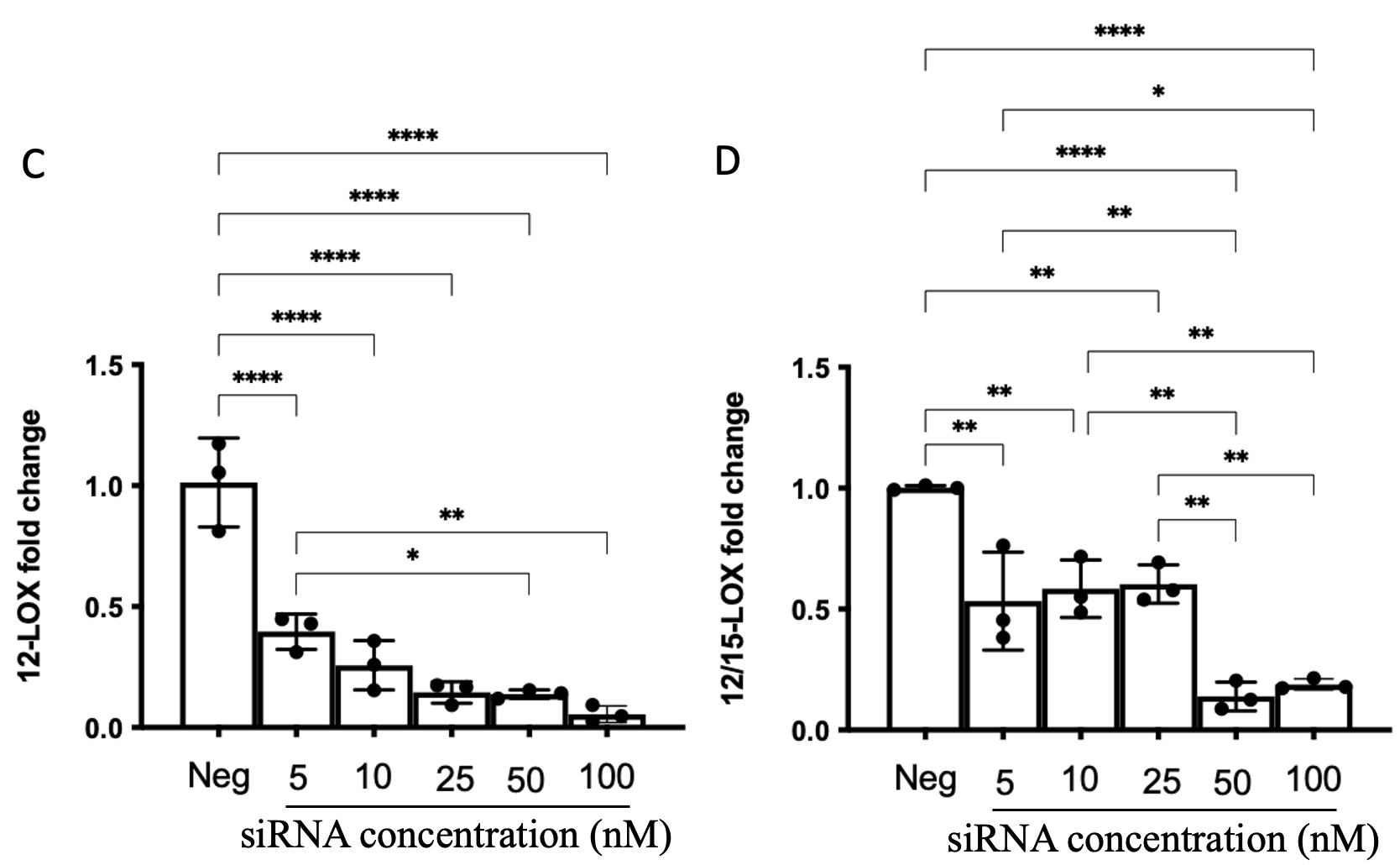


**S1 Fig:** **Optimization of siRNA concentrations**. RAW 264.7 cells were transfected separately with different siRNAs for 48 hours by using DharmaFECT 4 Transfection Reagent. (**A**) On-TARGETplus mouse GAPDH positive control and Non-Targeting Pool negative control siRNA were introduced at a final concentration of 25 nM. Total cellular RNA was isolated from transfected cells and subjected to RT-PCR analysis. mRNA expression of GAPDH in the positive control group was effectively silenced. (**B**) Western blot analysis of Tβ4 revealed a dose-dependent gene silencing, with the 50 nM Tβ4 siRNA concentration achieving sufficient interference. Transfection efficacies of 12-LOX (**C**) and 12/15-LOX (**D**) were assessed through RT-PCR, with relative transcription level changes across different concentrations quantified. When normalized to β-actin and presented with SD, both 12-LOX and 12/15-LOX showed significant transcriptional downregulation at the 50 nM concentration. Each RT-PCR experiment was repeated three times. * *p* < 0.05, ** *p* < 0.01, *** *p* < 0.001, **** *p* < 0.0001.


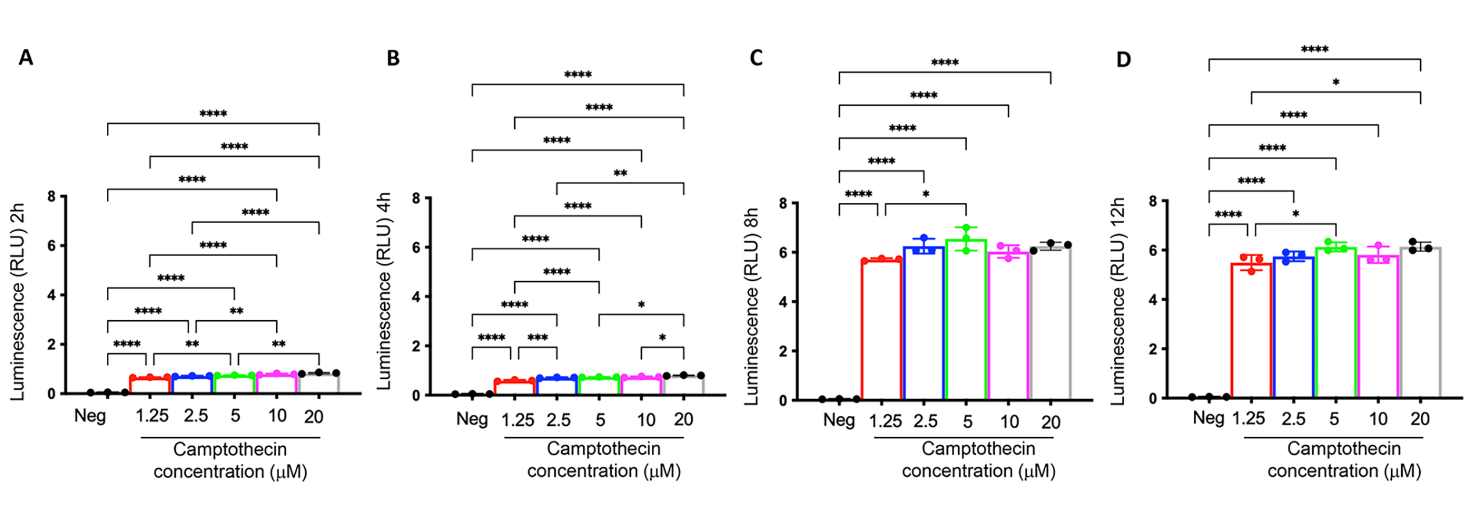


**S2 Fig:** **Determination of camptothecin concentration**. HL-60 cells underwent camptothecin treatment at increasing concentrations (0-20 μM) over varying durations: 2 h (**A**), 4 h (**B**), 8 h (**C**), and 12 h (**D**). Apoptotic responses were determined using an apoptosis assay, with luminescence measured at 485nm_Ex_ and 525nm_Em_ wavelengths. The no-cell, no-camptothecin group provided the background to account for the inherent luminescence of the reagents. By subtracting this baseline, net relative luminescent units were obtained. The pronounced induction of apoptosis was observed at both 8 h and 12 h, with 5 μM of camptothecin proving optimal. Data represents mean values from at least three independent experiments conducted in duplicate, accompanied by the SD. **p* < 0.05, ** *p* < 0.01, *** *p* < 0.001, **** *p* < 0.0001.
